# Whole-cell biocatalysis for hydrogen storage and syngas conversion to formate using a thermophilic acetogen

**DOI:** 10.1186/s13068-020-1670-x

**Published:** 2020-02-28

**Authors:** Fabian M. Schwarz, Volker Müller

**Affiliations:** Molecular Microbiology & Bioenergetics, Institute of Molecular Biosciences, Johann Wolfgang Goethe University, Max-von-Laue-Str. 9, 60438 Frankfurt am Main, Germany

**Keywords:** Carbon capture, Syngas, Whole-cell biocatalysis, Closed-batch fermentation, Hydrogen-dependent CO_2_ reductase, Formate dehydrogenase, Hydrogenase, Thermophiles, *Thermoanaerobacter kivui*

## Abstract

**Background:**

In times of global climate change, the conversion and capturing of inorganic CO_2_ have gained increased attention because of its great potential as sustainable feedstock in the production of biofuels and biochemicals. CO_2_ is not only the substrate for the production of value-added chemicals in CO_2_-based bioprocesses, it can also be directly hydrated to formic acid, a so-called liquid organic hydrogen carrier (LOHC), by chemical and biological catalysts. Recently, a new group of enzymes were discovered in the two acetogenic bacteria *Acetobacterium woodii* and *Thermoanaerobacter kivui* which catalyze the direct hydrogenation of CO_2_ to formic acid with exceptional high rates, the hydrogen-dependent CO_2_ reductases (HDCRs). Since these enzymes are promising biocatalysts for the capturing of CO_2_ and the storage of molecular hydrogen in form of formic acid, we designed a whole-cell approach for *T. kivui* to take advantage of using whole cells from a thermophilic organism as H_2_/CO_2_ storage platform. Additionally, *T. kivui* cells were used as microbial cell factories for the production of formic acid from syngas.

**Results:**

This study demonstrates the efficient whole-cell biocatalysis for the conversion of H_2_ + CO_2_ to formic acid in the presence of bicarbonate by *T. kivui*. Interestingly, the addition of KHCO_3_ not only stimulated formate formation dramatically but it also completely abolished unwanted side product formation (acetate) under these conditions and bicarbonate was shown to inhibit the membrane-bound ATP synthase. Cell suspensions reached specific formate production rates of 234 mmol g_protein_^−1^ h^−1^ (152 mmol g_CDW_^−1^ h^−1^), the highest rates ever reported in closed-batch conditions. The volumetric formate production rate was 270 mmol L^−1^ h^−1^ at 4 mg mL^−1^. Additionally, this study is the first demonstration that syngas can be converted exclusively to formate using an acetogenic bacterium and high titers up to 130 mM of formate were reached.

**Conclusions:**

The thermophilic acetogenic bacterium *T. kivui* is an efficient biocatalyst which makes this organism a promising candidate for future biotechnological applications in hydrogen storage, CO_2_ capturing and syngas conversion to formate.

## Background

**Fig. 1:**
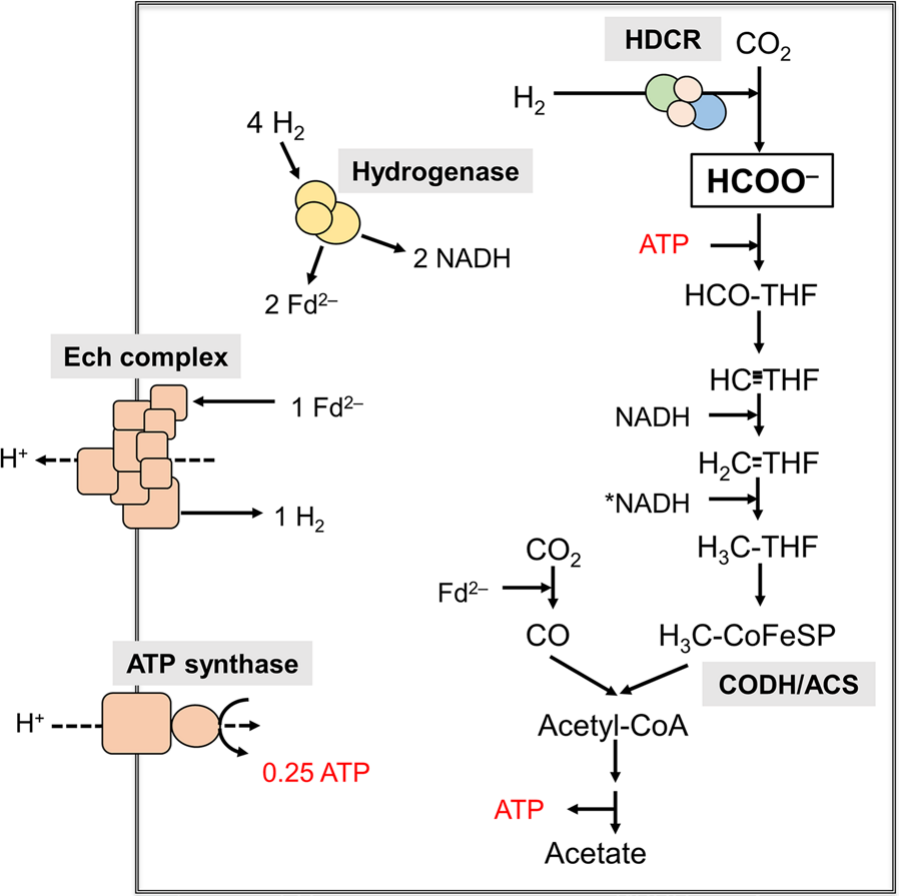
Model of the biochemistry and bioenergetics of acetogenesis from H_2_ + CO_2_ in *T. kivui*. The bioenergetics and biochemistry of acetogenesis from H_2_ + CO_2_ by *T. kivui* are shown. CODH/ACS, CO dehydrogenase/acetyl-CoA synthase; Ech, energy-conserving hydrogenase; HDCR, hydrogen-dependent CO_2_ reductase; hydrogenase, electron bifurcation hydrogenase; THF, tetrahydrofolic acid; HCO-THF, formyl-THF; HC-THF, methenyl-THF; H_2_C-THF, methylene-THF; H_3_C-THF, methyl-THF; CoFeSP, corrinoid iron–sulfur protein; Fd^2−^, reduced ferredoxin; * reduction of methylene-THF might occur using an electron donor with a similar redox potential as NADH

Carbon dioxide and syngas are considered as “renewable options” in biotechnological applications, especially in times of global climate change and gradual increase of atmospheric CO_2_ [1, 2]. Among the organisms able to reduce CO_2_, strictly anaerobic, acetogenic bacteria have gained much attraction in recent years [3–5] because they can use H_2_ and CO as reductant for CO_2_ reduction. The use of acetogenic bacteria to produce ethanol from syngas (H_2_, CO, CO_2_) is already realized on an industrial scale [6, 7]. The first step in acetogenic CO_2_ reduction is the reduction of CO_2_ to formic acid (Fig. 1). Acetogens are phylogenetically very diverse and employ different enzymes for this reaction [8, 9]. Typically, they have NADP- or ferredoxin-dependent formate dehydrogenases [10–12], whereas *Acetobacterium woodii* and *Thermoanaerobacter kivui* have a different enzyme, a hydrogen-dependent CO_2_ reductase (HDCR) [13, 14]. This enzyme has a formate dehydrogenase module and a [FeFe]-hydrogenase module that are connected by two small FeS-containing proteins. In contrast to formate dehydrogenases, these enzymes can use molecular hydrogen directly as reductant for CO_2_, without the need for external soluble cofactors. Interestingly, the enzyme also accepts electrons from CO (via ferredoxin) [14], making it a catalyst for the conversion of syngas to formic acid. The HDCR not only reduces CO_2_ with remarkable catalytic activities but also oxidizes H_2_ and, thus, can be used to kill two birds with one stone [14, 15]. Apart from CO_2_ reduction, it can be used to store hydrogen gas in a liquid, non-toxic product, formic acid or its base, formate, a so-called liquid organic hydrogen carrier (LOHC) [16, 17]. The equilibrium constant for the conversion of CO_2_ + H_2_ to formic acid is close to one and, therefore, it is an ideal biocatalyst for the storage of H_2_. All other enzymes known, including the membrane-bound formate hydrogen lyase of *Escherichia coli* have a strong bias towards formate oxidation and reduce CO_2_ only under harsh conditions with low activities [18, 19].

The isolated HDCR from *A. woodii* and *T. kivui* require strictly anoxic conditions which makes an application rather difficult. Using *A. woodii*, we have overcome this problem by establishing an efficient whole-cell system to convert H_2_ + CO_2_ to formic acid and vice versa [14, 15]. This system makes use of the ATP-dependent further conversion of formate in acetogens (Fig. 1). By lowering the cellular ATP content, formate is no longer reduced to acetate and stoichiometrically produced from H_2_ + CO_2_. However, *A. woodii* cannot grow on syngas or CO [20, 21] and resting cells produced only little formate from syngas and high amounts of acetate were still produced as unwanted side product [14]. In contrast, the HDCR containing thermophile *T. kivui* can grow in mineral medium on CO or syngas [22, 23]. Therefore, we started out to analyze hydrogenation of CO_2_ in a whole-cell system of *T. kivui* with the aim to increase productivity (due to its thermophilic nature) and to establish an efficient whole-cell biocatalyst for hydrogen storage and formate production from syngas.

## Results

### Formate production by *T. kivui* cells

**Fig. 2:**
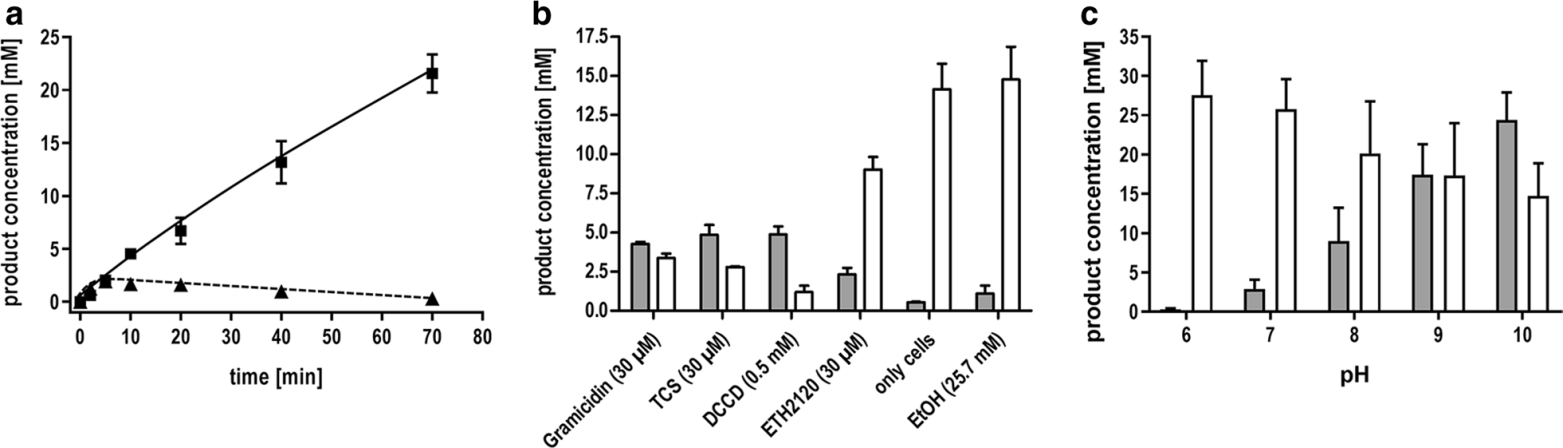
Effect of ionophores/uncoupling agents and pH on product formation by resting cells of *T. kivui*. Cells were grown with 0.1 M pyruvate, harvested in the end-exponential growth phase and suspended in buffer (50 mM Imidazole, 20 mM KCl, 20 mM MgSO_4_, 2 mM DTE, 4 µM Resazurin, pH 7.0 or 25 mM MES, 25 mM MOPS, 25 mM HEPES, 25 mM EPPS, 25 mM CHES, 20 mM KCl, 20 mM MgSO_4_, 2 mM DTE, 4 µM Resazurin, pH as indicated) to a final concentration of 1 mg/mL. **a** Cells were incubated without or **b** with ionophores/uncoupling agents or (**c**) at the corresponding pH for 10 min at 60 °C. The experiment was started by replacing the gas phase with H_2_ + CO_2_ (80:20%, 2 × 10^5^ Pa) and the product formation was determined after 40 (**b**) or 90 min (**c**). Squares and white bars, acetic acid; triangles and grey bars, formic acid

To analyze the potential use of whole cells of *T. kivui* as microbial cell factories for the efficient conversion of H_2_ + CO_2_ to formate, the organism was grown in complex medium with pyruvate as substrate and resting cells were prepared. As expected, the addition of H_2_ + CO_2_ to the cell suspension resulted in the production of acetate as the major end product with a specific acetate production rate of 19 mmol g_protein_^−1^ h^−1^ (12 mmol g_CDW_^−1^ h^−1^) (Fig. 2a). Formate was only produced in low amounts at the beginning of the experiment and was consumed afterwards. This is expected since formate is an intermediate in the WLP. As seen before with *A. woodii*, formate accumulation requires inhibition of further formate metabolism [14]. This can be achieved by reducing the energy status of the cell (Fig. 1). Hence, formate can no longer be activated due to a lack of ATP. One possibility to uncouple the energy metabolism of cells is by using ionophores. Depending on the ionophores used, there was a variation in the formate/acetate ratio after incubation with H_2_ + CO_2_ as substrate (Fig. 2b). In contrast to *A. woodii*, whose energy metabolism is strictly Na^+^ dependent [24, 25], the Na^+^ ionophore ETH2120 had almost no effect on product formation in *T. kivui* and the dominant compound was acetate. 9.1 mM acetate was produced but only 2.3 mM formate. This is consistent with previous experiments and the assumption that H^+^ instead of Na^+^ is used as the coupling ion for the primary bioenergetics in *T. kivui* [26, 27]. Thus, a more favorable formate to acetate ratio of 1.7 was achieved using the protonophore 3,3,4,5-tetrachlorosalicylanilide (TCS). A four times higher formate yield was detected using the ATPase inhibitor *N*,*N*′-dicyclohexylcarbodiimide (DCCD). Since acetate was still produced, the membrane potential seemed to be not fully diminished by the ionophores used in this study.

Interestingly, a change in pH had a dramatic effect on the product yields (Fig. 2c). At pH 6.0, there was no formate produced, but formate production increased with increasing pH. At the same time, acetate production decreased, but to a lesser extent. This led to an inversion of the formate/acetate ratio from 0.01 at pH 6.0 to 1.7 at pH 10.

**Fig. 3:**
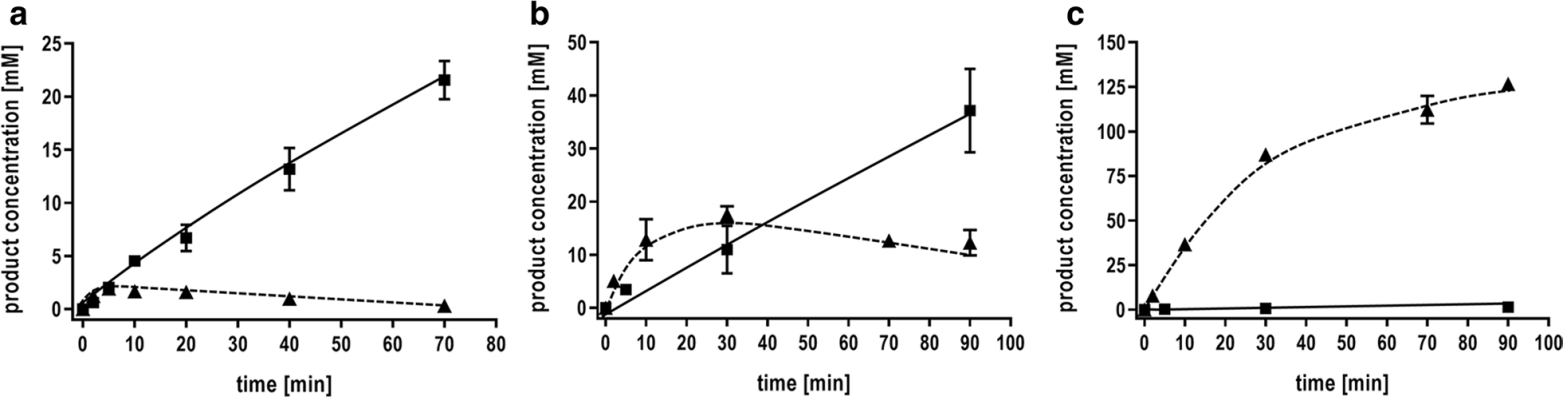
Effect of bicarbonate on product formation from H_2_ + CO_2_. **a** Resting cells of *T. kivui* (1 mg/mL) were incubated in anoxic buffer (50 mM Imidazole, 20 mM KCl, 20 mM MgSO_4_, 2 mM DTE, 4 µM resazurin, pH 7.0) with H_2_ + CO_2_ (80:20%, 2 × 10^5^ Pa). **b** 50 mM or **c** 300 mM KHCO_3_ were added to the cell suspensions before the experiment was started by replacing the gas phase. Squares, acetic acid; triangles, formic acid

For further experiments, we added bicarbonate to resting cells to increase the available amount of CO_2_ in solution and to achieve higher formate yields. At 50 mM bicarbonate, the acetate formation rate was slightly increased by 24% and, more important, the transient formation of formate was also increased by 319% (Fig. 3). At 300 mM bicarbonate acetate formation was completely abolished and formate production was drastically stimulated: The formate production rate was 220 mmol g_protein_^−1^ h^−1^ (143 mmol g_CDW_^−1^ h^−1^) and the final formate concentration reached 126 mM after 90 min.

### Inhibitory effect of bicarbonate on ATP synthesis

**Fig. 4:**
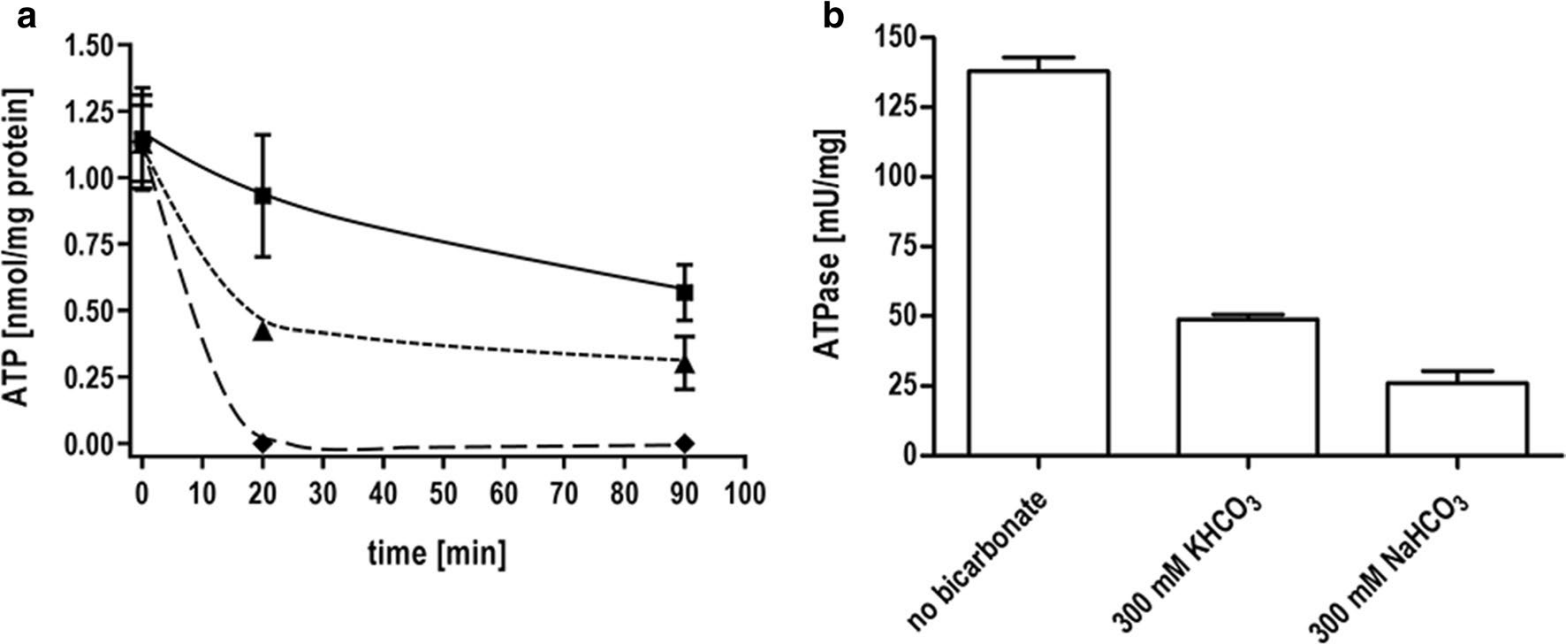
Effect of bicarbonate on the ATP content of resting cells and ATP hydrolysis catalyzed by membranes of *T. kivui*. **a** Resting cells of *T. kivui* (1 mg/mL) were incubated in anoxic buffer (50 mM Imidazole, 20 mM KCl, 20 mM MgSO_4_, 2 mM DTE, 4 µM Resazurin, pH 7,0) with H_2_ + CO_2_ (80:20%, 2 × 10^5^ Pa) in the absence or presence of KHCO_3_ and the ATP content of cells were determined. Squares, without bicarbonate; triangles, 50 mM KHCO_3_; diamonds, 300 mM KHCO_3_. **b** Membranes from *T. kivui* were incubated for 3 min in the presence (300 mM KHCO_3_ or 300 mM NaHCO_3_) or absence of bicarbonate in buffer (100 mM Tris/HCl, 20 mM MgSO_4_, 20 mM KCl, pH 7.0) and the ATP hydrolysis of membranes was determined

To analyze the effect of bicarbonate on the energy metabolism of *T. kivui* in detail, the cellular ATP content of resting cells was measured in the presence or absence of bicarbonate (Fig. 4a). Therefore, cells were incubated in buffer with H_2_ + CO_2_ as substrate, increasing bicarbonate concentrations were added and the ATP content was measured over time. As seen in Fig. 4a, the ATP content dropped immediately to zero if 300 mM bicarbonate was present in the cell suspensions. At 50 mM bicarbonate, there was also a decrease in the intracellular ATP content, but only by 62%. Next, we investigated the effect of bicarbonate on the activity of the membrane-bound ATPase in *T. kivui* (Fig. 4b). After the preparation of membranes, ATP hydrolysis was measured in the presence or absence of bicarbonate. Indeed, ATP hydrolysis as catalyzed by membranes (138 mU/mg) was inhibited by 81% by 300 mM NaHCO_3_. The same was observed with KHCO_3_. Additionally, we examined the ability of ATP synthesis by cell suspensions of *T. kivui* with an artificial ∆pH over the membrane as driving force (Additional file 1: Figure S1). In this experiment, resting cells were incubated in the presence or absence of 300 mM KHCO_3_ and then HCl was added to induce a ∆pH across the membrane. At a ∆pH of 6, ATP was synthesized to 3.2 nmol mg_protein_^−1^. In contrast, when cells were incubated with 300 mM KHCO_3_, ATP was only synthesized to 1.1 nmol mg_protein_^−1^. In accordance with the ATP hydrolysis experiments, only 34% of the ATP was synthesized in the presence of bicarbonate. Overall, these experiments could be interpreted to mean that the ATP synthase is inhibited by bicarbonate.

A possible pH effect by the addition of bicarbonate to the cell suspension was excluded. Therefore, the pH was adjusted in the control experiments with KOH to the same pH as in cell suspensions with additional 300 mM bicarbonate. The change in pH from 7.0 to 8.2 by the addition of KOH did not result in the same formate production. Only 14 mM of formate was formed after 90 min (data not shown).

### Characterization of hydrogen-dependent CO_2_ reduction by whole cells

**Fig. 5:**
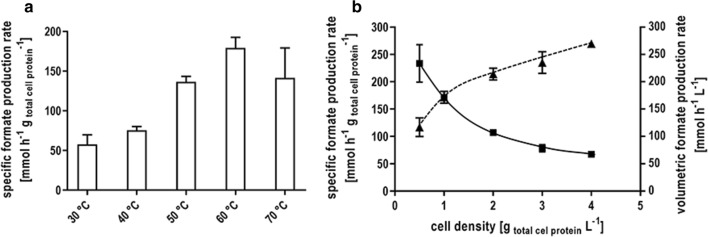
Characterization of hydrogen-dependent CO_2_ reduction by whole cells of *T. kivui*. **a** Resting cells of *T. kivui* (1 mg/mL) were incubated in anoxic buffer (50 mM Imidazole, 20 mM KCl, 20 mM MgSO_4_, 2 mM DTE, 4 µM Resazurin, pH 7.0) with H_2_ + CO_2_ (80:20%, 2 × 10^5^ Pa) in the presence of 300 mM KHCO_3_. Shown is the temperature profile for formate production by whole cells at the temperature indicated. **b** The influence of the cell density on formate production by resting cells was determined by applying a final concentration of 0.5–4 mg mL^−1^ in anoxic serum bottles at a temperature of 60 °C. 300 mM KHCO_3_ was added to the cell suspension and the experiments were started by replacing the gas phase with H_2_ + CO_2_ (80:20%, 2 × 10^5^ Pa). The initial formate production rates (squares) and the volumetric production rates (triangle) were plotted against the cell density used in the experiment

After the discovery that bicarbonate completely inhibits further downstream processing of formate, formate production from H_2_ + CO_2_ was studied in detail in the presence of 300 mM KHCO_3_. The cells showed highest specific formate production rates of 220 mmol g_protein_^−1^ h^−1^ (143 mmol g_CDW_^−1^ h^−1^) at a temperature of 60 °C (Fig. 5a). Nevertheless, even at moderate reaction temperature of 30 °C, there was still a catalytic activity of 58 mmol g_protein_^−1^ h^−1^ (38 mmol g_CDW_^−1^ h^−1^). Moreover, an increase of the specific formate production rate up to 234 mmol g_protein_^−1^ h^−1^ (152 mmol g_CDW_^−1^ h^−1^) was observed at a cell concentration of 0.5 mg mL^−1^ (Fig. 5b). Increasing cell densities resulted in a linear increase of the volumetric formate production rates up to 270 mmol L^−1^ h^−1^ at 4 mg mL^−1^. Simultaneously, the specific rates decreased.

*Thermoanaerobacter kivui* is a promising organism for industrial applications, since it can grow on syngas/CO in mineral medium without the requirement for yeast extract and additional vitamins [22, 28]. Therefore, we investigated the specific formate production rate of resting cells that were grown on mineral medium with pyruvate or glucose as growth substrate (Additional file 1: Figure S2). No differences in the specific formate production rates were observed if the complex medium was replaced by defined mineral medium in the cultivation process. Glucose-grown cells (in mineral medium) showed a slight decrease of 33% in the specific formate production rate compared to pyruvate grown cells.

### Syngas conversion to formate

**Fig. 6:**
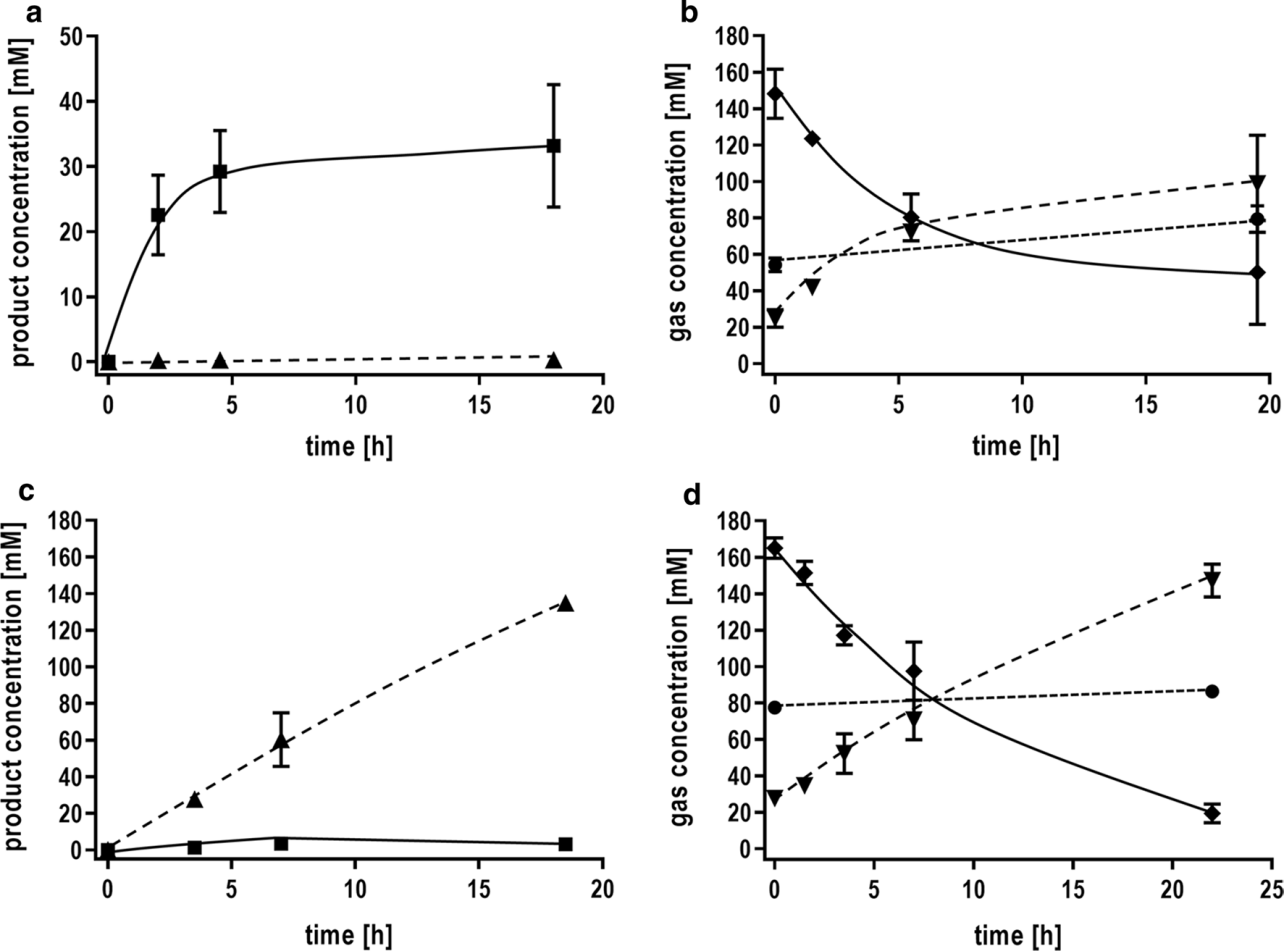
Formate production from syngas using whole cells of *T. kivui*. Cells were grown on 50% CO in complex medium, harvested in the end-exponential growth phase and suspended in buffer (50 mM Imidazole, 20 mM KCl, 20 mM MgSO_4_, 2 mM DTE, 4 µM Resazurin, pH 7,0) to a final concentration of 1 mg/mL in anoxic serum bottles. The experiment was started by replacing the gas phase with a mixture of H_2_ + CO_2_ + CO (26:11:63%, 2 × 10^5^ Pa) **a**, **b** in the absence of bicarbonate and **c**, **d** in the presence of 300 mM KHCO_3_. The product formation in liquid (**a**), **c** and the gas consumption in the head space (**b**, **d**) is shown over time. Squares, acetic acid; triangles, formic acid; diamonds, CO; triangles down, CO_2_; circles, H_2_

Syngas is an increasingly considered “green” option for the production of chemicals and biofuels [1] and *T. kivui* was already shown to grow on CO or syngas [22]. To analyze whether syngas is converted to formate, cells were grown on 50% CO and cell suspensions were prepared. A syngas mixture of H_2_ (26%), CO_2_ (11%) and CO (63%) was used as substrate. The gas consumption in the head space of the serum bottles was monitored by gas chromatography. In the absence of bicarbonate, resting cells converted syngas to acetate (Fig. 6a, b). Notably, the CO concentration decreased by 99 mM. At the same time, H_2_ and CO_2_ increased by only 26 and 74 mM, indicating that CO and H_2_ were used as reductant for CO_2_. If additional bicarbonate was added to the cell suspension, the product spectrum changed and mainly formate was produced in high titers up to 130 mM (Fig. 6c, d). The specific formate production rate was 8 mmol g_protein_^−1^ h^−1^ (5 mmol g_CDW_^−1^ h^−1^). CO was almost completely used up but the hydrogen level remained almost the same. This indicates that H_2_ is not oxidized in the presence of CO and an alternative electron donor seems to be used for the reduction of CO_2_ to formate. Additionally, a clear increase in the CO_2_ concentration was detectable, provoked by the interconversion of HCO_3_^−^ to CO_2_. This is the first demonstration that syngas can be converted exclusively to formate by an acetogenic bacterium. *T. kivui* cells which were not adapted on CO, instead grown heterotrophic with pyruvate as substrate, showed only a little formation of acetate and almost no formate was produced in the presence of bicarbonate.

### Formate production in closed-batch fermentation

**Fig. 7:**
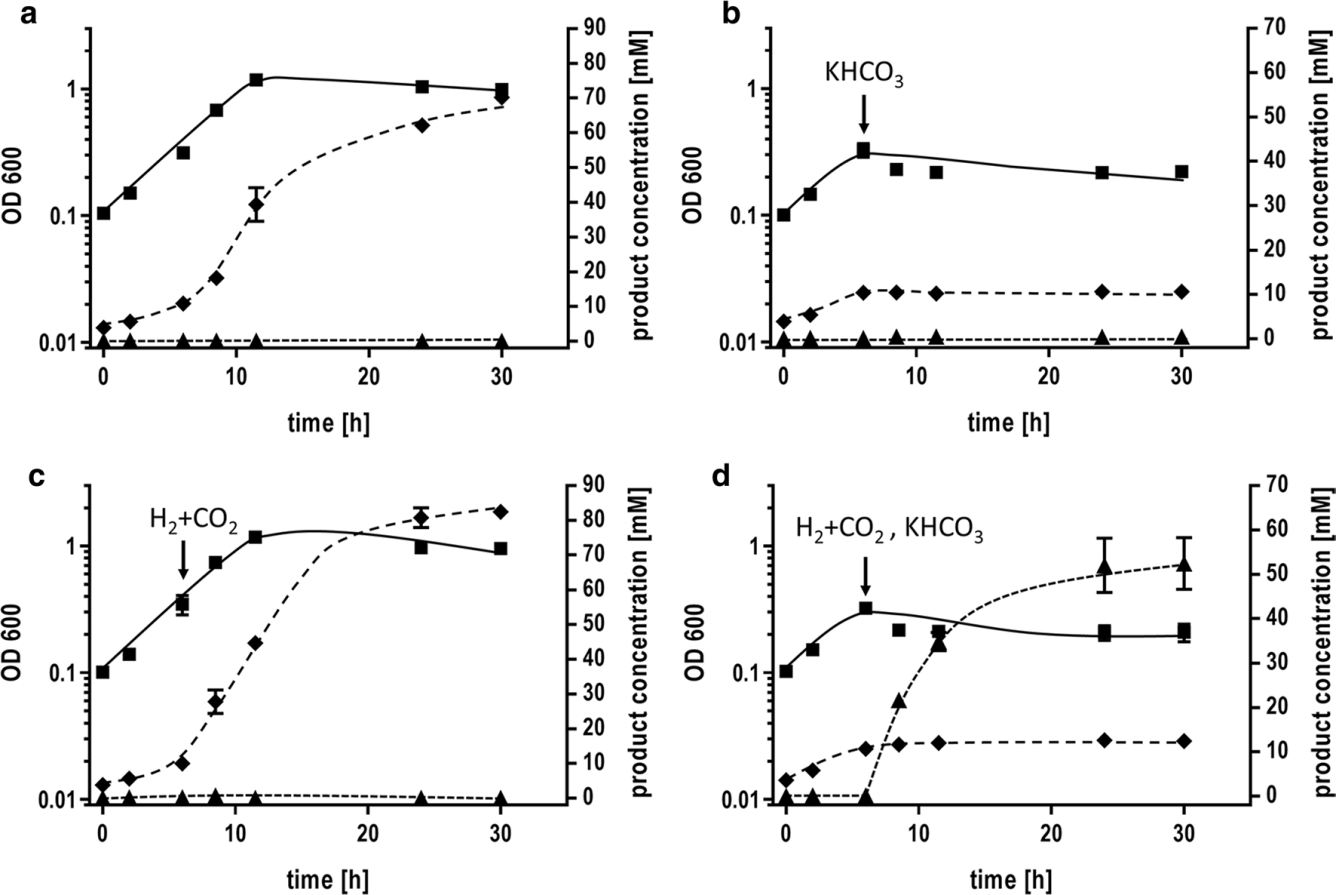
Closed-batch fermentation for hydrogen-dependent CO_2_ reduction to formic acid. **a***T. kivui* was grown on 28 mM glucose in a defined mineral medium in a shaking water bath at 66 °C. At the time point indicated **b** 300 mM KHCO_3_, **c** H_2_ + CO_2_ (80:20%, 2 × 10^5^ Pa) or **d** H_2_ + CO_2_ (80:20%, 2 × 10^5^ Pa) + 300 mM KHCO_3_ were added to the growing culture. The optical density of the culture was between 0.3 and 0.4. Squares, OD_600_; diamonds, acetic acid; triangles, formic acid

Next, we wanted to establish a production platform for formate in closed-batch fermentation (Fig. 7). Here, *T. kivui* cells were grown in defined mineral medium with 28 mM glucose as substrate (*t*_D_ = 3.2 h) to an optical density of ~ 0.3. Then, bicarbonate, H_2_ + CO_2_ or a combination of both were added. The addition of bicarbonate led to an immediate growth arrest and stop of acetate formation. By adding H_2_ + CO_2_, the optical density did not increase but cells produced more acetate. Formate was not produced overall. Now, when bicarbonate and H_2_ + CO_2_ were added, growth as well as acetate formation was completely abolished, but cells started to produce formate. The specific rate of formate production was 96 mmol g_protein_^−1^ h^−1^ (62 mmol g_CDW_^−1^ h^−1^). Finally, up to 50 mM formate was produced in the cultivation broth.

## Discussion

Resting cells of *T. kivui* were proven in this study as highly efficient whole-cell biocatalysts for the direct hydrogenation of CO_2_ to formate with remarkable catalytic activities. In addition, we showed the first whole-cell approach for the exclusive conversion of syngas to formate using an acetogenic bacterium. The recently identified hydrogen-dependent CO_2_ reductase (HDCR) [13] is the key enzyme in whole cell of *T. kivui* used as microbial cell factories for hydrogen storage, CO_2_ capturing and syngas conversion to formate.

Since the K value for Eq. 1

1 $$\text{H}_{2} + \text{CO}_{2} \mathop \Leftrightarrow \limits^{{}} \text{HCOO}^{ - } + \text{H}^{ + } \quad \quad \Delta G^{0'} = 3.5\; \text{kJ} \text{mol}^{ - 1}$$

is close to one, the chemical equilibrium can be easily controlled by small variations in pH, pressure and substrate/product concentrations. High concentrations of formate will favor the backwards reaction. An alkaline environment serves as proton scavenger and therefore pulls the reaction to the product side. The favored formate formation from H_2_ + CO_2_ in a more alkaline environment was also observed in a whole-cell system for hydrogen-dependent CO_2_ reduction based on *E. coli* [29]. By the addition of bicarbonate to resting cells, the available amount of CO_2_ in solution was increased and the reaction was pushed towards product formation. Since formate dehydrogenases of the Mo/W-bis PGD family are known to use only CO_2_ and not bicarbonate as substrate [12], we inspected the genome of *T. kivui* to identify a putative carbonic anhydrase (CA) which catalyzes the rapid interconversion of HCO_3_^−^ and CO_2_, and found one gene annotated as putative carbonic anhydrase/acetyltransferase (TKV_c11400). Consistent with this, cell extracts of *T. kivui* had specific CA activity of 0.17 U/mg [30].

Bicarbonate was identified here as an inhibitor of ATP synthesis. The inhibition of bacterial F_1_F_0_ ATP synthases by bicarbonate is not a common feature, but the effect was already described in literature [31, 32]. The effect of different anions like sulfite, azide and bicarbonate on the ATPase activity of membrane-bound F_1_F_0_ is known for decades but a detailed understanding of the mechanism of action of the activating anions is still missing and a matter of controversy [33–36]. In our study potassium bicarbonate could be replaced by sodium bicarbonate, indeed indicating an inhibitory effect of the anion HCO_3_^−^. Lodeyro et al. concluded in their study [32] that the anion bicarbonate competes with the binding of ADP to a low-affinity binding site instead of binding to a P_i_ site in the F_1_ subunit. They postulated that ATP hydrolysis and inhibition of ATP synthesis was affected by bicarbonate by modulating the relative affinities of the catalytic site for ATP and ADP. Since anions like bicarbonate and acid were shown to bind to different sites on the mitochondrial F_1_ subunit, further studies for the direct identification of the HCO_3_^−^ binding site on the F_1_F_0_ ATP synthase of *T. kivui* have to be done. Purification and characterization of this enzyme could help to finally elucidate the mechanism and site of action of bicarbonate.

Whole-cell biocatalysis for the production of formate from the greenhouse gas CO_2_ and the energy carrier H_2_ was also observed in other biological systems [14, 29, 37]. Besides the acetogenic bacteria *A. woodii* and *T. kivui*, the well-known model organism *E. coli* was also used as a cell factory for the hydrogenation of CO_2_ [29]. The key enzyme in *E. coli* to catalyze H_2_ + CO_2_ conversion to formate is the membrane-bound formate hydrogen lyase (FHL) complex [18, 38]. But this enzyme is designed by nature to produce H_2_ and CO_2_ from formate under fermentative conditions and therefore, the catalytic rates for formate formation are pretty low and harsh conditions are required for the reaction. In a pH-controlled and highly pressurized reactor system (up to 10 bar overpressure), the specific formate production rates were 15 mmol g_CDW_^−1^ h^−1^ [29]. This is only a small fraction of the activity of whole cells from *T. kivui* at moderate conditions of 30 or 60 °C with one bar overpressure. Here, the cells showed specific formate production rates of 58 mmol g_protein_^−1^ h^−1^ (38 mmol g_CDW_^−1^ h^−1^) and 220 mmol g_protein_^−1^ h^−1^ (143 mmol g_CDW_^−1^ h^−1^), respectively, qualifying *T. kivui* for applications at high and moderate reaction temperatures. Nevertheless, the thermophilic acetogenic bacterium *T. kivui* showed the highest specific formate production rates of 234 mmol g_protein_^−1^ h^−1^ (152 mmol g_CDW_^−1^ h^−1^) ever reported in biological systems (Table 1).

**Table 1:** Whole-cell biocatalysis for hydrogen-dependent CO_2_ reduction to formate in closed-batch conditions

Organism	Reaction condition: temperature (°C)	Reaction condition: overpressure (MPa)	Mode	Specific formate production rate (mmol g_CDW_^−1^ h^−1^)	Refs.
*E. coli* (WT)	37	10	Closed-batch bioreactor^a^	~15	[29]
*E. coli* (rec. strain^b^)	37	−	Closed-batch (flasks)	~0.1	[37]
*Desulfovibrio desulfuricans* (WT)	37	1	Closed-batch (flasks)	~0.7	[39]
*A. woodii* (WT)	30	1	Closed-batch (flasks)	~22	[14]
*T. kivui* (WT)	30	1	Closed-batch (flasks)	~38	This study
	60	1	Closed-batch (flasks)	~152	This study

*WT* wild-type strain

^a^pH-controlled

^b^Rec. strain, recombinant *E. coli* strain JM109(DE3) overexpressing FDH of *Pyrococcus furiosus* (*FDH_*Pyrfu)

Furthermore, the volumetric formate production rates of 270 mmol L^−1^ h^−1^ at cell concentrations of 4 mg mL^−1^ is not an insignificant economical factor: implementing high cell densities in a later fermentation process is considered to be one of the most effective ways for enhancing the productivity [40]. Efficient cell recycling and cell retention systems with optimized conditions for the accumulation of high cell densities up to 200 g/L were already implemented in bioprocesses [41–43].

The fermentation of syngas into biofuels and biochemicals using acetogenic bacteria has attracted more and more interest over the last few years and some acetogens were already implemented in this process [44–47]. Since the syngas composition depends strongly on the kind of gasifier and the kind and condition of the feedstock used, there is no “universal” composition of syngas. But it was already shown that *T. kivui* can be adapted to a carboxydotrophic lifestyle by a stepwise adaptation on increasing CO concentrations, up to 100% CO [22]. A detailed understanding of the CO metabolism in *T. kivui* is still missing. Since CO is a potent inhibitor of the active site of [FeFe]-hydrogenases [48–50], the HDCR hydrogenase subunit should be inactive and no formate should be formed. The inhibitory effect of CO on the HDCR hydrogenase activity of *A. woodii* was already described [14]. However, reduced ferredoxin can serve as an alternative electron donor for the reduction of CO_2_ to formate in in vitro studies. This correlates with the finding that H_2_ was not utilized by *T. kivui* in the previous syngas experiment if CO was present but formate was still produced. Therefore, the two annotated CO dehydrogenases genes in the genome of *T. kivui* could play a key role in the oxidation of CO to CO_2_ with simultaneous reduction of ferredoxin, which is subsequently used by the HDCR for a ferredoxin-driven CO_2_ reduction to formate.

In this study, we showed the feasibility of two approaches for the efficient conversion of H_2_ + CO_2_ to formate: whole-cell biocatalysis and closed-batch bioprocess/fermentation. But the production rates as well as the finally produced formate concentration differed between the two approaches. The reasons could be diverse and are probably linked to pH, buffer capacity, feedback inhibition, etc. The applicability of growing cells as microbial cell factories has to be proven in further fermentation studies. Nevertheless, the addition of bicarbonate and H_2_ + CO_2_ can switch the growing culture to the production of formate instead of acetate. The gases H_2_ + CO_2_ can also serve in the first phase as growth substrate till the production phase is initiated. In this production phase, H_2_ + CO_2_ act as reactants for the efficient production of formate. Whether the minimized cost-intensive and time-consuming work flow in a closed-batch fermentation can rebalance the increasing downstream costs due to the accumulation of unwanted metabolic side products (e.g., acetate) in the fermentation broth during the growth phase has to be considered and individually calculated.

## Conclusion

This work demonstrates an efficient whole-cell approach for the production of formate from H_2_ + CO_2_ or syngas using the thermophilic acetogen *T. kivui*. Bicarbonate seems to be an efficient inhibitor of the ATP synthase of this organism, thus preventing further downstream conversion of formate to acetate, resulting in high titers of the desired end product. *T. kivui* catalyzed the hydrogen-dependent CO_2_ reduction with remarkable catalytic activities at elevated and ambient temperatures. Its thermophilic nature and the autotrophic growth properties on mineral medium qualify this organism for future fermentation approaches to address the process on a larger scale and to investigate the stability of the whole-cell system.

## Methods

### Organism and cultivation

*Thermoanaerobacter kivui* LKT-1 (DSM 2030) was cultivated at 66 °C under anaerobic conditions in complex and defined mineral medium [22]. Media were prepared under anoxic conditions as described before [51, 52]. Glucose (28 mM), pyruvate (100 mM) or CO (50% CO, 40% N_2_ and 10% CO_2_ [v/v] at 2 × 10^5^ Pa) were used as growth substrate. Cell were cultivated in 1-L flasks (Müller-Krempel, Bülach, Switzerland) containing 500 mL or 200 mL medium in the case of autotrophic cultivation. Growth was determined by measuring the optical density at 600 nm with an UV/Vis spectrophotometer.

### Preparation of resting cells and cell suspension experiments

For the preparation of resting cells, *T. kivui* was cultivated in 1-L flasks (Müller-Krempel, Bülach, Switzerland) in the above-mentioned growth media to the late exponential growth phase. Glucose- and fructose-grown cells were harvested at an OD_600_ of 1.7–2.0, CO-grown cells were harvested at OD_600_ of 0.6. The culture was centrifuged under anoxic conditions at 11,500 g and 4 °C for 10 min and was washed twice in imidazole puffer (50 mM imidazole–HCl, 20 mM MgSO_4_, 20 mM KCl, 2 mM DTE, 4 µM resazurin, pH 7.0). Afterwards, the cells were resuspended, if not otherwise stated, in the same buffer to a protein concentration of 1 mg/mL and kept in gas-tight Hungate tubes. All preparation steps were performed under strictly anoxic conditions at room temperature in an anaerobic chamber (Coy Laboratory Products, Grass Lake, MI) as described [53]. The protein concentration of the cell suspension was determined according to [54] and the cells were directly used for the subsequent cell suspension experiments.

To determine the conversion of H_2_ + CO_2_ in cell suspension experiments, the 120-mL serum flasks (Glasgerätebau Ochs GmbH, Bovenden-Lenglern, Germany) contained pre-warmed buffer under a N_2_ atmosphere, incubated with cell suspensions for 10 min at 60 °C. Subsequently, bicarbonate (KHCO_3_ or NaHCO_3_) was added and the gas phase of the serum flasks was changed to 2 × 10^5^ Pa H_2_ + CO_2_ (80:20 [v/v]). When syngas was the substrate, the reaction was started by replacing the head space of the serum flasks with a gas composition of 26% H_2_ + 11% CO_2_ + 63% CO [v/v] at 2 × 10^5^ Pa. Ionophores and uncoupling agents such as 3,3,4,5-tetrachlorosalicylanilide (TCS, dissolved in EtOH), *N*,*N*,*N*′,*N*′-tetracyclohexyl-1,2-phenylenedioxydiacetamide (ETH2120, dissolved in EtOH), gramicidin (dissolved in EtOH) and *N*,*N*′-dicyclohexylcarbodiimide (DCCD, dissolved in EtOH) were added 10 min prior to the reaction start. The serum flasks contained a final volume of 10 mL buffer in all the experiments. Samples were taken and ATP [55], acetate, formate, H_2_, CO_2_ and CO were determined as described before [13, 22].

### Preparation of membranes and measurement of ATP hydrolysis activity

Cells were grown in 500 mL complex medium in 1-L flasks (Glasgerätebau Ochs, Bovenden-Lenglern, Germany) with 100 mM pyruvate as carbon source to an optical density at 600 nm of 1.7–2.0. The cells were harvested under toxic conditions at 11,500*g* for 10 min at 4 °C, were washed twice in buffer A (50 mM imidazole–HCl, 20 mM MgSO_4_, 20 mM KCl, pH 7.0) and membranes were prepared as described before [26]. The protein concentration was determined as described [56] and the membranes were directly used to measure ATP hydrolysis.

For the determination of the ATP hydrolysis, membranes (200 µg) were resuspended in buffer B (100 mM Tris/HCl, 20 mM MgSO_4_, 20 mM KCl, pH 7.0) to a final volume of 1200 µL and incubated at 60 °C for 3 min in the presence or absence of 300 mM KHCO_3_. After addition of 2.5 mM Na_2_ATP, samples (200 µL) were taken at defined time points and the ATP content was determined as described [55].

### Closed-batch fermentation

*Thermoanaerobacter kivui* was grown at 66 °C in 50 mL mineral medium in 120 mL serum flasks (Glasgerätebau Ochs GmbH, Bovenden-Lenglern, Germany) with 28 mM glucose as growth substrate and a gas phase of N_2_ + CO_2_ (80:20 [v/v]). At OD_600_ 0.3–0.4 the growing cells were switched into the formate production phase by addition of 300 mM KHCO_3_ and by changing the gas phase to a H_2_ + CO_2_ (80:20% [v/v]) atmosphere. Samples for the product determination were taken with a syringe.

### Determination of cell dry weight

For cell dry weight determination of *T. kivui*, three independent cultures were grown in complex medium with 0.1 M pyruvate as growth substrate. At three different optical densities in the exponential growth phase the culture was harvested (4150*g*, 30 min, 4 °C) in technical triplicates (3 × 50 mL). Afterwards, the cell pellet was frozen in liquid N_2_ and dried by lyophilisation over 24 h. The dried samples were weighted and the cell dry weight (CDW) was calculated to 0.379 mg/mL at OD_600_ of 1.

## Supplementary information

**Additional file 1: Figure S1.** ATP synthesis by cell suspensions of *T. kivui* driven by an artificial ∆pH. Cells were grown with 0.1 M pyruvate, harvested in the end-exponential growth phase and suspended in buffer (50 mM Imidazole, 20 mM KCl, 20 mM MgSO_4_, 2 mM DTE, 4 µM Resazurin, pH 7.0). Cell suspensions (1 mg/mL) were incubated with and without KHCO_3_ for 10 min in buffer (25 mM Tris/HCl, 20 mM MgCl_2_, pH 9.0) at 60 °C. At the time point indicated (arrow), HCl was added to the cell suspensions. Shown are data from one representative experiment out of two independent replicates. Squares, without KHCO_3_; triangles, 300 mM KHCO_3_.

**Additional file 2: Figure S2.** Specific formate production rates of resting cells from *T. kivui* grown on mineral medium. Cells were grown with 28 mM glucose or 0.1 M pyruvate in a defined mineral or complex medium, harvested in the end-exponential growth phase and suspended in buffer (50 mM Imidazole, 20 mM KCl, 20 mM MgSO_4_, 2 mM DTE, 4 µM Resazurin, pH 7.0) to a final concentration of 1 mg/mL in anoxic serum bottles. The bottles were incubated in a shaking water bath for 10 min at 60 °C with additional 300 mM KHCO_3_. The experiment was started by replacing the gas phase with H_2_ + CO_2_ (80:20%, 2 × 10^5^ Pa). *MM* mineral medium, *CM* complex medium.

## Data Availability

All data generated or analyzed during this study are included in this published article.

## Abbreviations

LOHC: Liquid organic hydrogen carrier
HDCR: Hydrogen-dependent CO_2_ reductase
*A. woodii*: *Acetobacterium woodii*
*T. kivui*: *Thermoanaerobacter kivui*
CDW: Cell dry weight
FHL: Formate hydrogen lyase
*E. coli*: *Escherichia coli*
ETH2120: *N*,*N*,*N*′,*N*′-Tetracyclohexyl-1,2-phenylenedioxydiacetamide
TCS: 3,3,4,5-Tetrachlorosalicylanilide
DCCD: *N*,*N*′-Dicyclohexylcarbodiimide
MES: 2-Morpholin-4-ylethanesulfonic acid
MOPS: 3-Morpholinopropane-1-sulfonic acid
HEPES: 2-[4-(2-Hydroxyethyl)piperazin-1-yl]ethanesulfonic acid
EPPS: 4-(2-Hydroxyethyl)piperazine-1-propanesulfonic acid
CHES: 2-(Cyclohexylamino)ethanesulfonic acid
Tris: 2-Amino-2-(hydroxymethyl)propane-1,3-diol
DTE: Dithioerythritol
HCO_3_^−^: Bicarbonate
*t*_D_: Doubling time
*K*: Equilibrium constant
$$\Delta G^{{0\varvec{'}}}$$: Gibbs energy
Mo/W-bis PGD: Molybdenum/tungsten-bis pyranopterin guanosine dinucleotide
CA: Carbonic anhydrase
UV/Vis: Ultraviolet/visible
OD: Optical density
