# Supplementary figures and images for: Whole-cell biocatalysis for hydrogen storage and syngas conversion to formate using a thermophilic acetogen
Source: Biotechnol Biofuels. 2020 Feb 28;13:32. doi: 10.1186/s13068-020-1670-x (PMC7048051; doi:10.1186/s13068-020-1670-x)

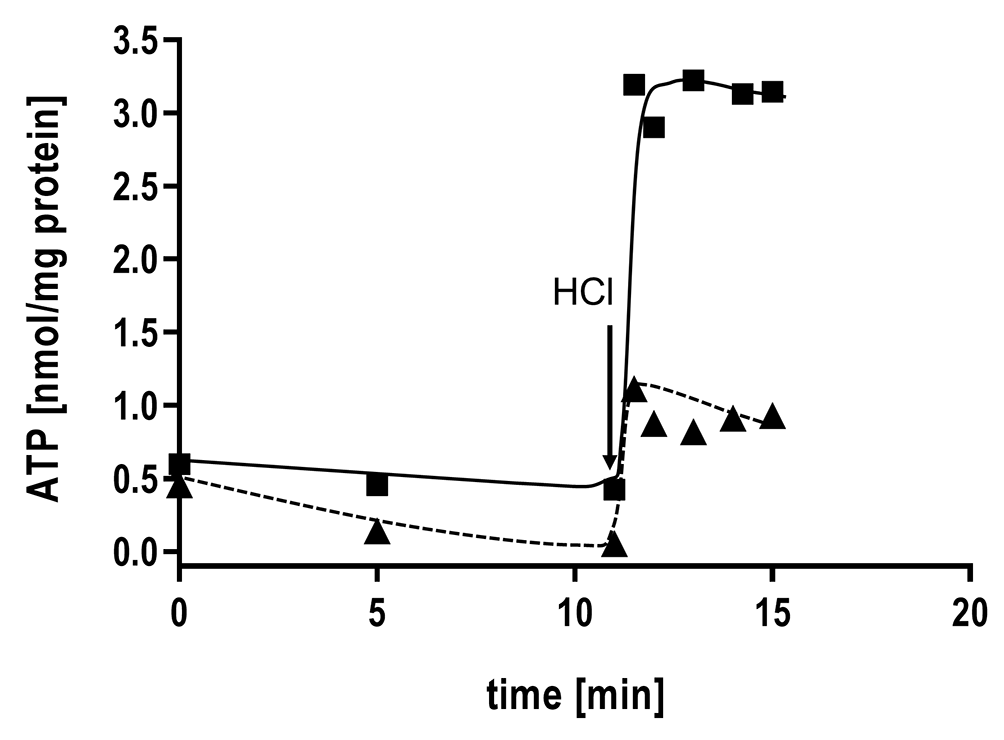

Supplement: Supplementary file 1 — Additional file 1: Figure S1. ATP synthesis by cell suspensions of T. kivui driven by an artificial ∆pH. Cells were grown with 0.1 M pyruvate, harvested in the end-exponential growth phase and suspended in buffer (50 mM Imidazole, 20 mM KCl, 20 mM MgSO4, 2 mM DTE, 4 µM Resazurin, pH 7.0). Cell suspensions (1 mg/mL) were incubated with and without KHCO3 for 10 min in buffer (25 mM Tris/HCl, 20 mM MgCl2, pH 9.0) at 60 °C. At the time point indicated (arrow), HCl was added to the cell suspensions. Shown are data from one representative experiment out of two independent replicates. Squares, without KHCO3; triangles, 300 mM KHCO3. [file 13068_2020_1670_MOESM1_ESM.tif]

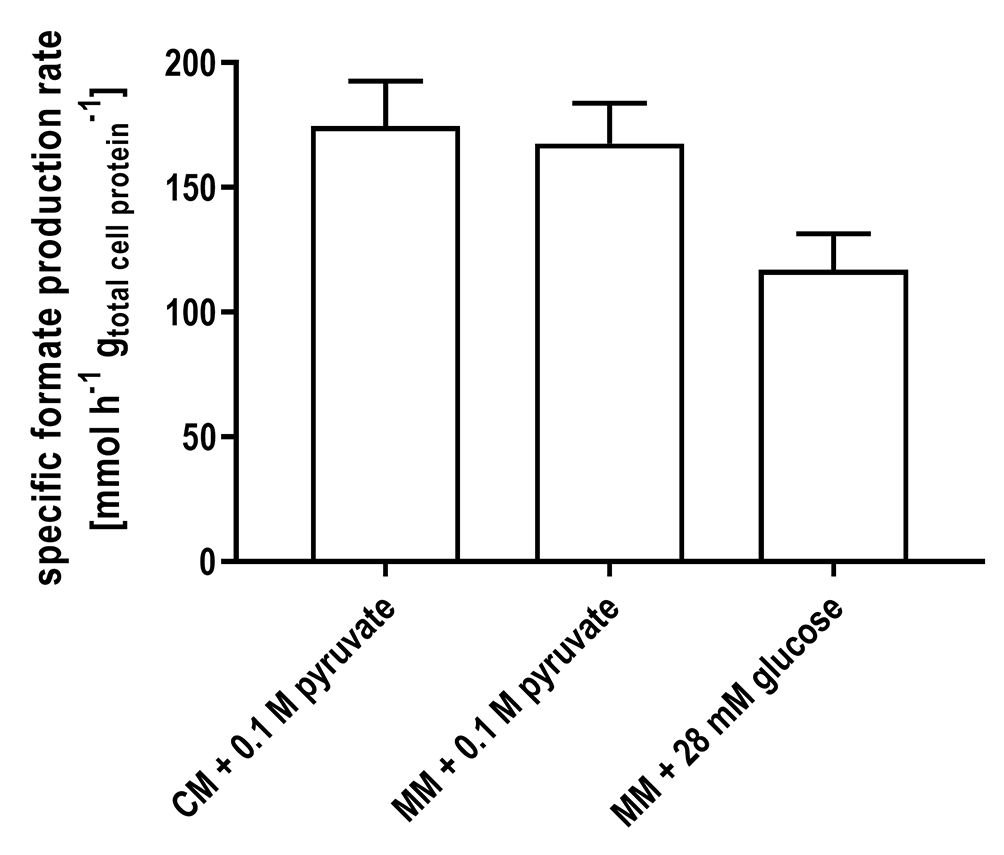

Supplement: Supplementary file 2 — Additional file 2: Figure S2. Specific formate production rates of resting cells from T. kivui grown on mineral medium. Cells were grown with 28 mM glucose or 0.1 M pyruvate in a defined mineral or complex medium, harvested in the end-exponential growth phase and suspended in buffer (50 mM Imidazole, 20 mM KCl, 20 mM MgSO4, 2 mM DTE, 4 µM Resazurin, pH 7.0) to a final concentration of 1 mg/mL in anoxic serum bottles. The bottles were incubated in a shaking water bath for 10 min at 60 °C with additional 300 mM KHCO3. The experiment was started by replacing the gas phase with H2 + CO2 (80:20%, 2 × 10^5^ Pa). MM mineral medium, CM complex medium. [file 13068_2020_1670_MOESM2_ESM.tif]
